# Systematic review to estimate the prevalence of inflammatory rheumatic diseases in Germany

**DOI:** 10.1007/s00393-022-01302-5

**Published:** 2023-02-07

**Authors:** Katinka Albrecht, Sebastian Binder, Kirsten Minden, Denis Poddubnyy, Anne C. Regierer, Anja Strangfeld, Johanna Callhoff

**Affiliations:** 1 Programme Area Epidemiology and Health Services Research, German Rheumatism Research Centre Berlin, Charitéplatz 1, 10117 Berlin, Germany; 2 Department of Pediatric Respiratory Medicine, Immunology and Critical Care Medicine, Charité Universitätsmedizin Berlin, Berlin, Germany; 3 Department of Gastroenterology, Infectiology and Rheumatology, Charité Universitätsmedizin Berlin, Berlin, Germany; 4 Department of Rheumatology and Clinical Immunology, Charité Universitätsmedizin Berlin, Berlin, Germany; 5 Institute for Social Medicine, Epidemiology and Health Economics, Charité Universitätsmedizin Berlin, Berlin, Germany

**Keywords:** Rheumatoid arthritis, Spondyloarthritis, Juvenile idiopathic arthritis, Connective tissue diseases, Vasculitis, Rheumatoide Arthritis, Spondylarthritis, Juvenile idiopathische Arthritis, Kollagenosen, Vaskulitis

## Abstract

**Objective:**

This study aimed to update the prevalence estimates of inflammatory rheumatic diseases (IRD) in Germany.

**Methods:**

A systematic literature search in PubMed and Web of Science (last search 08 November 2022) identified original articles (regional and nationwide surveys and claims data analyses for arthritides, connective tissue diseases, and vasculitides) on prevalences for the period 2014–2022. Data sources, collection period, case definition, and risk of bias are reported. Prevalences were estimated from available national data, with consideration of international data.

**Results:**

Screening by two authors yielded 263 hits, of which 18 claims data analyses and 2 surveys met the inclusion criteria. Prevalences ranged from 0.42 to 1.85% (rheumatoid arthritis), 0.32–0.5% (ankylosing spondylitis), 0.11–0.32% (psoriatic arthritis), 0.037–0.14% (systemic lupus erythematosus), 0.07–0.77% (Sjögren’s disease/sicca syndrome), 0.14–0.15% (polymyalgia rheumatica, ≥ 40 years), 0.04–0.05% (giant cell arteritis, ≥ 50 years), and 0.015–0.026% (ANCA-associated vasculitis). The risk of bias was moderate in 13 and high in 7 studies. Based on the results, we estimate the prevalence of IRD in Germany to be 2.2–3.0%, which corresponds to approximately 1.5–2.1 million affected individuals. The prevalence of juvenile idiopathic arthritis was reported to be around 0.10% (0.07–0.10%) of 0–18-year-olds, corresponding to about 14,000 children and adolescents in Germany.

**Conclusion:**

This systematic review shows an increase in the prevalence of IRD in Germany, which is almost exclusively based on claims data analyses. In the absence of multistage population studies, the available data are, overall, uncertain sources for prevalence estimates, with a moderate to high risk of bias.

**Supplementary Information:**

The online version of this article (10.1007/s00393-022-01302-5) contains supplementary material.

## Abstract (de)

**Zielsetzung:**

Ziel der Arbeit war eine aktualisierte Schätzung der Prävalenz entzündlich-rheumatischer Erkrankungen (ERE) in Deutschland.

**Methodik:**

Mit einer systematischen Literaturrecherche in den Datenbanken PubMed und Web of Science (letzte Suche am 08.11.2022) wurden Originalartikel (regionale und bundesweite Surveys und Routinedatenanalysen für Arthritiden, Kollagenosen und Vaskulitiden) zur ERE-Prävalenz für den Zeitraum 2014–2022 identifiziert. Datenquellen, Erhebungszeitraum, Diagnosedefinition und das Risiko einer Verzerrung werden mitgeteilt. Die Prävalenzen wurden anhand der verfügbaren nationalen Daten unter Berücksichtigung internationaler Angaben geschätzt.

**Ergebnisse:**

Die Suche durch 2 Autorinnen ergab 263 Treffer, von denen 18 Routinedatenanalysen und 2 Surveys die Einschlusskriterien erfüllten. Die Prävalenzangaben lagen bei 0,42–1,85 % (rheumatoide Arthritis), 0,32–0,5 % (ankylosierende Spondylitis), 0,11–0,32 % (Psoriasisarthritis), 0,037–0,14 % (systemischer Lupus erythematodes), 0,07–0,77 % (Sjögren‑/Sicca-Syndrom), 0,14–0,15 % (Polymyalgia rheumatica, ab 40 Jahre), 0,04–0,05 % (Riesenzellarteriitis, ab 50 Jahre) und 0,015–0,026 % (mit antineutrophilen zytoplasmatischen Antikörpern [ANCA-]assoziierte Vaskulitis). Das Bias-Risiko war in 13 Studien moderat, in 7 Studien hoch. Anhand dieser Ergebnisse schätzen die Autorinnen die Prävalenz von ERE in Deutschland auf 2,2–3,0 %. Dies entspricht in etwa 1,5–2,1 Mio. Betroffenen. Die Prävalenz der juvenilen idiopathischen Arthritis wurde mit etwa 0,10 % (0,07–0,13 %) der 0‑ bis 18-Jährigen angegeben, was etwa 14.000 Kindern und Jugendlichen in Deutschland entspricht.

**Schlussfolgerung:**

Dieses systematische Review zeigt einen Anstieg der Prävalenzen von ERE in Deutschland, basiert jedoch fast ausschließlich auf Routinedatenanalysen. In Ermangelung mehrstufiger Bevölkerungsstudien sind die vorliegenden Daten bei moderatem bis hohem Verzerrungsrisiko insgesamt unsichere Quellen für Prävalenzschätzungen.

For health care planning in rheumatology, there is a need for current estimates on the prevalence of inflammatory rheumatic diseases in Germany. In addition to actual changes in the frequency of the diseases and the ageing of the German population, also improved early diagnosis and a decline in mortality may have led to an increase in prevalence rates in recent years. As there is no population register for inflammatory rheumatic diseases in Germany, the prevalence can only be estimated using the available national data and, in case of missing data, using European or other international publications. In 2016, we calculated the prevalence and distribution of musculoskeletal diseases based on the available evidence [1]. This paper presents a systematic review to identify newly available evidence up to 2022. Frequencies were recalculated to match the current German population figures.

## Methods

A systematic literature search (SLR) was conducted in the PubMed and Web of Science databases for the period from January 2014 (end of the previous period [1]) to October 2022 (last search on 08 November 2022). Original studies published in German or English that investigated the prevalence of inflammatory rheumatic diseases (arthritides, connective tissue diseases, vasculitides) in adults or children and adolescents in Germany were included. The following search terms were used: prevalence, rheumatoid arthritis, spondyloarthritis, spondylitis, lupus erythematosus, polymyalgia rheumatica, Sjögren’s disease, inflammatory myositis, ANCA-associated vasculitis, rheumatic disease. The search strategy and process are presented in the supplementary material. The literature was selected by two persons (KA, JC) independently of each other. In case of disagreement, a consensus was reached. In addition, a search was conducted in the references of the selected publications by asking experts for further studies and by internet research. Of 263 articles, 16 met the inclusion criteria and 4 were supplemented by manual search. Included studies were assigned to the following clinical pictures: rheumatoid arthritis (RA), axial spondyloarthritis (axSpA) or ankylosing spondylitis (AS), psoriatic arthritis (PsA), systemic lupus erythematosus (SLE), Sjögren’s disease, polymyalgia rheumatica (PMR), giant cell arteritis (GCA), antineutrophil cytoplasmic antibodies (ANCA)-associated vasculitis, and juvenile idiopathic arthritis (JIA).

The included articles were checked for risk of bias using a checklist adapted from Hoy et al. [2]. For each study, four aspects of external validity and five aspects of internal validity were checked, and each was assessed on two levels (high or low risk of bias). In the overall judgement formed from these assessments, the risk of bias was classified as high, moderate, or low. A meta-analysis based on SLR was deliberately not carried out, as the studies found are not comparable in terms of many criteria and various methodological prerequisites for a meta-analysis are not fulfilled. For classification of the data from Germany and the estimates derived from them, we additionally consulted European literature, systematic reviews, and German reviews, but these are not part of the SLR. The paper was written in accordance with the Preferred Reporting Items for Systematic Reviews and Meta-Analyses (PRISMA) reporting guidelines [3].

## Results

With the SLR, 18 claims data analyses and 2 surveys were identified. The prevalence data and the respective data sources are reported in Table 1.

**Table 1:** Included articles from the systemic literature search on the prevalence of inflammatory rheumatic diseases in Germany

Reference	Diagnosis	Data source	Study population	Diagnosis definition	Investigation period	Prevalence, raw data	Prevalence in women/men	Standardization
Schmidt 2020 [4]	RA AS SLE Sjögren	Nationwide NAKO health study	101,779 responders (20–69 years)	Patient-reported medical diagnosis (ever)	2014–2017 (prevalence estimated from age- and sex-stratified random sample; one-time survey during the period)	RA: 1.85% AS: 0.49% SLE: 0.14% Sjögren: 0.07%	RA: 2.62%/1.08% AS: 0.42%/0.55% SLE:	Age and gender standardized to German standard population 2011
Kienitz 2020 [5]	RA	Nationwide SHI claims data	Approx. 2.3 million insured persons ≥ 18 years	(1) ICD: M05, M06; (2) Specialist diagnosis (3) ICD code + DMARD	2008–2013 2013 (annual prevalence in the respective annual cross section)	(1) 2008–2013: 1.17–1.34% (2) 2011–2013: 0.94–1.07% (3) 2008–2013: 0.44–0.54%	(1) 1.8%/0.8% (2013)	No standardization
Grellmann 2020 [6]	RA PsA	Nationwide SHI claims data	965,759–1930,158 (different in the years considered) ≥ 18 years	RA: M05.8, M06.0, M06.8 PsA: M07.0–3, L40.5	2012–2016 (annual prevalence in the respective annual cross section)	RA: 0.42–0.53% (2012–2016) PsA: 0.27–0.32%	Women of childbearing age: RA: 0.2% PsA: 0.1–0.2%	Age- and gender-standardized to the total SHI population in the respective year
Beam 2018 [7]	RA	Regional AOK claims data	3,446,670 insured persons	(1) ICD: M05, M06 (2) + Medication	2013	(1) 1.05% (2) 0.64%	(1): 1.4%/0.64% (2) 0.86%/0.39%	Age-standardized to “old” European standard population of 1976
Steffen 2017 [8]	RA	Nationwide SHI claims data	60–61 million insured persons	(1) M05, M06 + laboratory (2) 2014: at least 1 × ICD-code + laboratory in the entire period	2009–2015 (annual prevalence in the respective annual cross section)	(1) 2009: 0.87% (0.87%) (1) 2015: 1.08% (1.06%) (2) 2014: 1.23% (1.20%)	1.49%/0.62% (2015)	Age- and gender-standardized to SHI total population 2016
Hense 2016 [9]	RA	Nationwide BARMER claims data	7,155,315 insured persons	(1) M05, M06 (2) + Laboratory (3) + Medication (4) + Rheumatology	2013 (annual prevalence)	(1) 1.62% (1.38%) (2) 1.11% (0.95%) (3) 0.94% (0.81%) (4) 0.64% (0.55%)	–	Age and gender standardized to German standard population 2013
Krüger 2018 [10]	AS	Nationwide SHI claims data (InGef)	3.2 million insured persons	M45	2013 (annual prevalence)	322/100,000	–	Database representative of German population by gender and age, therefore no separate standardization
Deike 2021 [11], Sewerin 2019 [12]	PsA	Nationwide SHI claims data	64–65 million insured persons	Not specified	2009–2012 (annual prevalence in the respective annual cross section)	2009: 0.20% 2012: 0.24%	0.21–0.25%/ 0.18–0.21%	No standardization for total estimators
Reinhardt 2021 [13]	PsA Juvenile PsA	DAK claims data	2,319,584 insured persons	M07.0–3, M09.0 (juvenile)	2010 (annual prevalence)	0.31% (0.29%) Juvenile: 0.01% (0.01%)	–	Age- and gender-standardized to SHI total population 2012
Sondermann 2018 [14]	PsA	Regional AOK claims data	Approx. 2.8 million insured persons	L40.5	2014 (quarters 1 and 2)	0.11%	–	No standardization
Rech 2020 [15]	PsA	Nationwide claims data (InGef)	2.9 million adult insured persons	M07.0, M07.1, M07.3	2012–2017 (cumulated)	2017: 0.15%	–	Database assumed to be representative of German population by gender and age, therefore no separate standardization
Schwarting 2021 [16]	SLE	Nationwide BKK claims data	4.1 million adult insured persons	M32.1,8,9 + Laboratory/medication/specialist diagnosis	2009–2014 (annual prevalence in the respective annual cross section)	2009: 37.3 (38.6)/100,000 2014: 47.4 (48.5)/100,000 with statistical adjustment for right-censored data in 2014: 55.8/100,000	2014: 79.8/13.8 per 100,000	–
Brinks 2014 [17]	SLE	Nationwide SHI claims data	2.3 million insured persons	M32	2002 (annual prevalence)	36.7 [34.3–39.3]/100,000	55.4/15.4 per 100,000	No standardization
Albrecht 2020 [18]	Sjögren	Nationwide BARMER claims data	7.2 million insured persons ≥ 18 years	M35.0	2007–2018 (annual prevalence in the respective annual cross section)	2007–2018: 0.68–0.77%	0.87–0.97%/0.38–0.44% per 100,000	No standardization
Colombo 2022 [19]	PMR	Regional AOK claims data	Not specified ≥ 40 years	M35.3, M31.5	2011–2019 (annual prevalence in the respective annual cross section and cumulative)	2011:115(107)/100,000 2019: 153(145)/100,000 Cumulated: 139(130)/100,000	166/86 per 100,000 (cumulative, age standardized)	Age- and gender-standardized to total SHI population 2019
Herlyn 2014 [20]	GCA AAV	Regional survey	469,000 inhabitants	GCA: M31.5, M31.6 GPA: M31.3, EGPA: M30.3, MPA: M31.7 + CHCC definition, ACR criteria	2006 (annual prevalence)	GCA: 440 [399;481]/1 mio ≥ 50 years AAV: 149 [126;174]/1 mio GPA: 98 [79;117], MPA: 28 [18;117], EGPA: 24 [14;35]	GCA: 612/219 AAV: 271/328 per 1 m ≥ 50 years	No standardization on standard population
Hellmich 2021 [21]	AAV	Nationwide SHI claims data (InGef)	Approx. 3 million insured persons ≥ 18 years	M31.3 (GPA), M31.7 (MPA)	2013–2016 (cumulated)	AAV: 256 ± 11/1 mio GPA: 210 ± 7/1 mio MPA: 46 ± 4/1 mio	–	Database assumed to be representative of German population by gender and age, therefore no separate standardization
Thomschke 2018 [22]	JIA	Nationwide SHI claims data	Approx. 12 million insured persons 0–19 years	M08.–, M09.0 (L40.5)	2009–2015 (annual prevalence in the respective annual cross section)	2009: 73.4/100,000 until 2015: 101.5/100,000	119.8/58.9 per 100,000 (average annual prevalence)	No standardization
Luque Ramos 2017 [23]	JIA	Nationwide BARMER claims data	238,000 insured persons 16–18 years	M08.x, M09.0	2008–2010	2008: 0.11% 2009, 2010: 0.13%	–	No standardization

*AAV* antineutrophil cytoplasmic antibodies (ANCA)-associated vasculitis, *AS* ankylosing spondylitis, *SHI* statutory health insurance, *GPA *granulomatous polyangiitis, *JIA* juvenile idiopathic arthritis, *MPA* microscopic polyangiitis, *PMR* polymyalgia rheumatica, *PsA* psoriatic arthritis, *RA* rheumatoid arthritis, *GCA* giant cell arteritis, *SLE* systemic lupus erythematosus

### Assessment of the risk of bias

The risk of bias is listed in Table 2. All studies have a moderate to high risk of bias in the overall assessment. In terms of external validity, some studies have good representativeness for the national population and had a low risk of bias here. In two regional analyses, the risk was rated as high. There is no response bias in the claims data; this only applied to the surveys and was high in these studies due to a low response rate. Concerning internal validity, no direct survey was carried out for any of the claims data analyses; this is only the case for the surveys. Regarding the case definition, low risk was assumed for the claims data analyses if several case definitions were tested or additional criteria for inclusion were considered.

**Table 2:** Assessment of the risk of bias, checklist according to Hoy et al. [2]

*External validity* 1 Was the target population of the study a good representation of the national population in terms of the relevant variables? 2 Was the sampling frame a true or accurate representation of the target population? 3 Was a form of random selection used to select the sample OR was a census conducted? 4 Was the probability of non-response bias minimal? *Internal validity* 5 Were the data collected directly from the subjects (as opposed to a proxy)? 6 Did the study use an acceptable case definition? 7 Was the study instrument used to measure the parameter of interest valid and reliable? 8 Was the same type of data collection used for all subjects? 9 Was the length of the shortest prevalence period appropriate for the parameter of interest? 10 Were the numerator(s) and denominator(s) appropriate for the parameter of interest? 11 Summary item on overall risk of study bias											
–	1	2	3	4	5	6	7	8	9	10	11 (overall assessment)
Schmidt [4]	Low	Low	Low	High	Low	Low	High	Low	Low	Low	Moderate
Kienitz [5]	Low	Low	Low	Low	n.a.	Low	High	Low	Low	Low	Moderate
Grellmann [6]	Low	Low	Low	Low	n.a.	High	High	Low	Low	Low	Moderate
Beam [7]	High	High	Low	Low	n.a.	Low	High	Low	Low	Low	High
Steffen [8]	Low	Low	Low	Low	n.a.	Low	High	Low	Low	Low	Moderate
Hense [9]	High	Low	Low	Low	n.a.	Low	High	Low	Low	Low	Moderate
Krüger [10]	Low	Low	Low	Low	n.a.	Low	High	Low	Low	Low	Moderate
Deike [11]	Low	Low	Low	Low	n.a.	High	High	Low	Low	Low	Moderate
Sewerin [12]	Low	Low	Low	Low	n.a.	High	High	Low	Low	Low	Moderate
Reinhardt [13]	High	High	Low	Low	n.a.	High	High	Low	High	High	High
Sondermann [14]	High	High	Low	Low	n.a.	High	High	Low	Low	Low	High
Rech [15]	Low	Low	Low	Low	n.a.	High	High	Low	Low	Low	Moderate
Schwarting [16]	High	Low	Low	Low	n.a.	Low	High	Low	Low	Low	Moderate
Brinks [17]	Low	Low	Low	Low	n.a.	High	High	Low	Low	Low	Moderate
Albrecht [18]	High	Low	Low	Low	n.a.	High	High	Low	Low	Low	High
Colombo [19]	High	Low	Low	Low	n.a.	High	High	Low	Low	Low	High
Herlyn [20]	High	High	High	High	Low	Low	Low	High	Low	Low	High
Hellmich [21]	Low	Low	Low	Low	n.a.	Low	High	Low	Low	Low	Moderate
Thomschke [22]	Low	Low	Low	Low	n.a.	High	High	Low	Low	Low	Moderate
Luque Ramos [23]	High	Low	Low	Low	n.a.	High	High	Low	Low	Low	High

Two-stage assessment of the individual criteria: low or high; three-stage overall assessment: low, moderate, or high

*n.a.* not applicable

### Study results on prevalence and classification

#### Rheumatoid arthritis

**Fig. 1:**
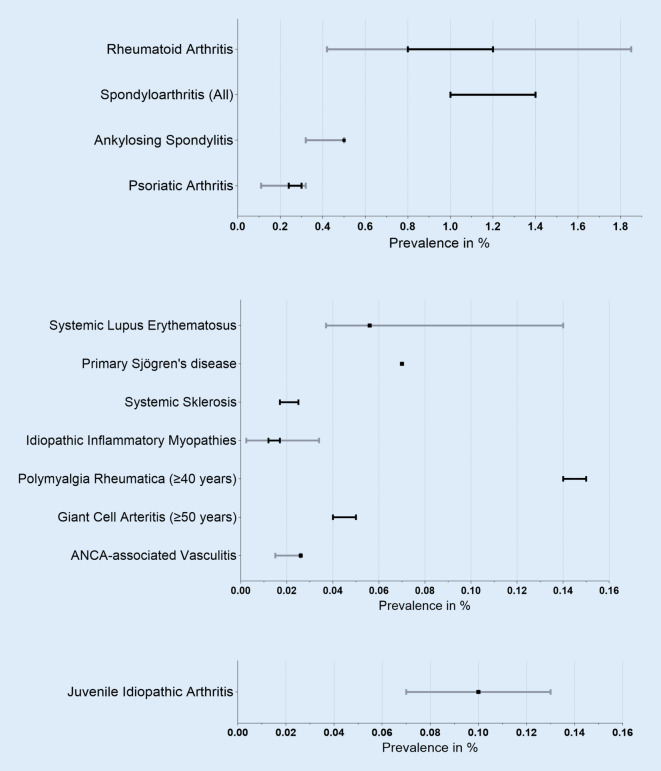
Prevalence of inflammatory rheumatic diseases from the systematic literature search (*grey*) and prevalence assumptions after data analysis (*black*)

For rheumatoid arthritis (RA), five claims data analyses and patient-reported data from the German National Cohort (NAKO) study are available. The prevalence data vary depending on the case definition of RA from 0.4% (Grellmann et al. [6]) to 1.85% (self-reported medical diagnosis from the NAKO [4]); see Fig. 1). The prevalence in Grellmann et al. is lower than in the other claims data analyses because only the specific ICD-10-GM codes for RA M05.8, M06.0, and M06.8 were included. However, in real-world care, the non-specific RA ICD-10-GM codes M06.9 and M05.9 are most commonly used [24], so an underreporting of RA cases can be assumed in this work. In the self-reports of the respondents from the NAKO study, overreporting is possible due to frequent use of the term “polyarthritis or rheumatism” even in cases of hand osteoarthritis and gout; here, it is difficult to differentiate in the context of a survey, although RA or polyarthritis were explicitly asked for. We also assume that the ICD-10-GM codes M05 and M06 are over-recorded, since M06.9 in particular is often coded when (any) inflammatory rheumatic disease is present. Multiple coding, e.g., RA (M06) and PsA, is frequent, and often, if the suspicion of RA is not confirmed, the M06 code is not deleted again (assessment from practice). The prevalence estimates that seem most plausible to us are those where in addition to ICD coding, a specific medication, a laboratory test of inflammatory markers, or a specialist diagnosis was required [5, 7–9]. Based on this type of case definition, we estimate that the prevalence of RA in the German adult population ranges from 0.8 to 1.2% [25]. Based on a population of 69.4 million adults in 2021 [26], this equates to approximately 560,000 to 830,000 persons currently affected (Table 3).

**Table 3:** Estimate of the prevalence of inflammatory rheumatic diseases in Germany

	Prevalence data (%) from the studies	Prevalence assumption (%) after analysis of the studies	Estimated number of people affected^a^	Accuracy of the estimate from the authors’ point of view
Rheumatoid arthritis	0.42–1.85	0.8–1.2	560,000–830,000	Moderate
Spondyloarthritis (All)	1.0–1.4^f^	1.0–1.4	690,000–970,000	Low
Ankylosing spondylitis	0.32–0.5	0.5	350,000	Low
Psoriatic arthritis	0.11–0.32	0.24–0.32	170,000–220,000	Moderate
Systemic lupus erythematosus	0.037–0.14	0.056	39,000	Moderate
Sjögren’s (sicca syndrome) Of which primary Sjögren’s disease	0.07–0.77	0.4–0.7 0.07	280,000–490,000 49,000	Low
Systemic sclerosis	0.017–0.025^f^	0.017–0.025	12,000–17,000	Low
Idiopathic inflammatory myopathies	0.0024–0.034^f^	0.012–0.017 (adults + children)	10,000–14,000^b^	Low
Total connective tissue diseases^g^	–	0.16–0.17	111,000–118,000	Low
Polymyalgia rheumatica	0.14–0.15 (≥ 40 years)	0.14–0.15 (≥ 40 years)	66,000–71,000^c^	Low
Giant cell arteritis	0.04–0.05 (≥ 50 years)	0.04–0.05 (≥ 50 years)	15,000–19,000^d^	Low
ANCA-associated vasculitides	0.015–0.026	0.026	18,000	Moderate
Inflammatory rheumatic diseases in adults	–	2.2–3.0	Approx. 1.5–2.1 million adults	Moderate
Juvenile idiopathic arthritis	0.07–0.13	0.10	Approx. 14,000 children and young people^e^	Moderate

^a^Based on 69.4 million adults

^b^83.2 million adults and children and adolescents

^c^47.5 million adults ≥ 40 years

^d^37.5 million adults ≥ 50 years

^e^13.9 million children and adolescents < 18 years in the German population in 2021 [26]

^f^International or older data from Germany

^g^Systemic lupus erythematosus, primary Sjögren’s disease, systemic sclerosis, and myopathies

#### Axial spondyloarthritis

There are few data from Germany on the frequency of axial spondyloarthritis (axSpA). In the NAKO study, 0.49% reported ankylosing spondylitis (AS) [4]. However, the frequencies reported from the NAKO study cannot be considered accurate prevalence estimates: the response rate in the survey was very low at 18%, and the cohort only included people aged 20–69 years; thus, selection bias is likely. A claims data analysis using the Institute for Applied Health Research (InGef) database estimated a prevalence of ICD-10-GM M45 diagnosis of 0.32% in 2013, which was used to extrapolate the number of adults with AS in Germany to 217,400 [10]. The proportion of 0.31% with an M45 diagnosis in 2020 was comparable in a BARMER claims data analysis, which was, however, not primarily aimed at estimating prevalence [27]. Whether the ICD-10-GM M45 diagnosis covers all persons with AS remains uncertain. In the Norwegian patient registry, the prevalence of axSpA was 0.41% in the adult population in 2017 [28]. In addition to AS, axSpA also includes nonradiological forms, which significantly increases the number of people affected. There is no current study on this from Germany. Based on older data from the 1998 Berlin study, the prevalence of AS in Germany has been estimated at 0.5% and that of SpA as a whole at 1–1.4% [29]. This is in good agreement with prevalence estimates from the USA (AS 0.52–0.55%, axSpA 1–1.4% [30]) and would correspond to approximately 350,000 persons with AS and approximately 690,000–970,000 persons with SpA in Germany.

#### Psoriatic arthritis

Five claims data analyses are available for the prevalence estimate of psoriatic arthritis (PsA). In the study from Grellmann et al. [6], the annual prevalence for the years 2012–2016 was between 0.27% and 0.32%, using the ICD-10-GM codes M07.0–3 and L40.5 as diagnostic criteria. The prevalence of Reinhardt et al., with 0.29% in 2010 using similar diagnostic criteria, is comparable [13]. Sewerin et al. have estimated, based on the diagnosis prevalence of 0.21% (men) and 0.25% (women) from 2012, that there were at least 200,000 affected persons in Germany in 2018 [12]. With the same data basis, the prevalence for 2012 was reported by Deike et al. as 0.24% [11]. Using data from InGef, Rech et al. identified 4390 persons with PsA in a collective of 2.8 million insured persons in 2012. This corresponds to a prevalence of 0.15%, whereby Rech et al. did not include the code M07.2 (spondylitis psoriatica) [15]. Likewise, the prevalence of 0.11% reported by Sondermann et al. seems to be an underestimate, as only the code L40.5 in two quarters from the year 2014 was considered [14]. If international data are included, the pooled prevalence of PsA in a meta-analysis including 28 studies was 0.13% (95% confidence interval [CI]: 0.11–0.16% [31])—with great variability among the individual studies and significantly lower than the prevalence from the Norwegian patient register of 0.46% in the adult population [31]. Two studies with psoriasis collectives from Germany showed after clinical examination that 20% of psoriasis patients also had PsA; many of them were undiagnosed [32, 33]. In another study from 2014, the proportion of diagnosed PsA in the psoriasis population studied was already 19% and a further 11% were newly diagnosed by rheumatological examination [34]. It can therefore be assumed that the proportion of PsA in psoriasis could be significantly higher than previously described [1]. Since undiagnosed cases are not included in routine health insurance data and the diagnosis code is more specific than the ICD-10-GM M06 RA code, we tend to assume an underestimation of case numbers in the claims data. Therefore, we estimate a prevalence of 0.24–0.32% for PsA in Germany, which would correspond to approximately 170,000–220,000 affected persons.

### Connective tissue diseases

#### Systemic lupus erythematosus

For systemic lupus erythematosus (SLE), two claims data analyses and data from the NAKO study were included. Using the prevalence of SLE diagnosis in 2002 of 36.7 (34.3–39.3)/100,000, Brinks et al. projected the number of people with SLE to be 31,000 in 2010, with a further increase by 2020 [17]. Schwarting et al. found the prevalence for 2014 to be even higher, at 55.8/100,000 [16], although in this study, an SLE-specific diagnostic procedure, medication, or specialist diagnosis was required for inclusion. International data present large variability, with overall lower prevalence data in Europe compared to the USA [35]. The estimated prevalence from the UK was 97/100,000 in 2012, markedly higher than that from Germany, and showed an increase compared to the years prior to 2012 [36]. In the NAKO study, 0.14% of respondents reported ever having been diagnosed with SLE by a physician [4]. It remains uncertain whether cutaneous forms were also marked as SLE by the respondents and this number should therefore be classified as an overestimate. If we match the results to the international data, a prevalence of approximately 0.056% seems plausible, which would correspond to about 39,000 affected persons.

#### Sjögren

A claims data analysis showed a high prevalence of the ICD-10-GM diagnosis of Sjögren’s syndrome (M35.0: sicca syndrome [Sjögren’s syndrome]) between 0.68% and 0.77% from 2007–2018 [18]. The ICD-10 diagnosis does not distinguish between primary and secondary forms. The classification in ICD-10 is made under “Other diseases with systemic involvement of connective tissue,” so that only primary and secondary forms in connective tissue diseases are coded under this and not those that occur in other diseases. However, the age and gender distributions of Sjögren’s coded in the claims data do not correspond to the distribution expected based on clinical experience. Therefore, we suspect a clear overestimation of the real prevalence, with overly frequent coding due to sicca symptoms. For Germany, a prevalence of at least 0.4% has been assumed so far, including secondary forms [37]. In the NAKO study, 0.07% of respondents reported Sjögren’s syndrome [4], which may more likely correspond to primary Sjögren’s, but it was not asked specifically about primary or secondary forms. The global prevalence of primary Sjögren’s is reported to be 60.8 (95%CI [43.7–77.9])/100,000, with a higher prevalence in Europe [38]. We estimate the proportion of individuals with primary Sjögren’s to be around 0.07% and the proportion including secondary forms to be 0.4–0.7%—with great uncertainty regarding the differentiation from sicca symptoms in noninflammatory diseases. This would correspond to approximately 49,000 (primary Sjögren’s) or 280,000–490,000 (primary and secondary) affected persons.

There are no studies from Germany on systemic sclerosis and idiopathic inflammatory myopathies, so we report international data.

#### Systemic sclerosis

For systemic sclerosis, there are relatively consistent prevalence data from Sweden from 2015 of 22.7/100,000 [39], from Denmark with 17.9–19.2/100,000 in the years 2009–2016 [40], and from Great Britain from 17.1–25.4/100,000 in the years 2000–2012 [41]. These correspond well with unpublished data from the German Network for Systemic Scleroderma (DNSS), which suggest an approximate prevalence of 20/100,000 (communication Prof. Blank, Heidelberg). We therefore use a range of 17–25/100,000 for an estimate, which would correspond to approximately 12,000–17,000 adults.

#### Idiopathic inflammatory myopathies

The prevalence of idiopathic inflammatory myopathies was 14 (95%CI 13–15)/100,000 in the Swedish Patient Registry in 2012, which included dermatomyositis, polymyositis, inclusion body myositis, juvenile dermatomyositis, and non-specific myositis. When a broader or stricter case definition was chosen, the prevalence was 12 (95%CI 11–13) to 17 (95%CI 16–18)/100,000 [42]. International prevalences from a systematic review ranged from 2.4 to 33.8/100,000 [43]. In the absence of national data, we use the range of 12–17/100,000 from the Swedish study for an estimate, which would correspond to approximately 10,000–14,000 affected persons (including children and adolescents with juvenile myositis) in a population of 83.2 million.

If one adds up the prevalence data of the connective tissue diseases (SLE, primary Sjögren’s, and international data on systemic sclerosis and myositides), one arrives at an approximate prevalence of 0.16–0.17%, which would correspond to approximately 111,000–118,000 affected persons.

#### Polymyalgia rheumatica

For polymyalgia rheumatica (PMR), only one analysis is available with data from the AOK Württemberg, which calculated an age- and sex-standardized prevalence of PMR of 145 (95%CI: 143–147)/100,000 in persons aged 40 years and older for 2019 [19]. The prevalence was not calculated for people over 50 years of age, so we cannot use these data to determine an estimate for the classification-appropriate age group of 50 years and older. International data are much higher, with prevalence estimates of 370–850/100,000 for people aged 50 and over [19, 44]. If we take the figures from Baden-Württemberg as a basis, this would correspond to 47.5 million adults ≥ 40 years of age in 2021 [26] and approximately 66,000–71,000 affected persons.

#### Giant cell arteritis

The prevalence of giant cell arteritis (GCA) was investigated in a regional survey in Lübeck and Bad Segeberg (both towns in the area of Schleswig-Holstein in northern Germany) in 1994 and 2006. In 1994, the prevalence was 24 (95%CI:14–35)/100,000 and in 2006, 44 (95%CI: 40–48)/100,000 for persons aged 50 years and older [20]. In the international context, these numbers are in the lower range: an international systematic review reported very heterogeneous prevalences from 20 (95%CI: 16–24)/100,000 (Turkey) to 250 (95%CI: 110–390)/100,000 (UK), in each case related to adults aged 50 years and older [44]. If we extrapolate the data from Schleswig-Holstein, this would correspond to approximately 15,000–19,000 affected persons in 37.5 million adults ≥ 50 years.

#### ANCA-associated vasculitides

For ANCA-associated vasculitides (AAV), as with GCA, the regional survey by Herlyn et al. showed a doubling of the prevalence from 74/1 million in 1994 to 149 (95%CI:126–174)/1 million in 2006 [20]. Based on the ICD-10-GM diagnosis codes, claims data from InGef from the years 2013–2016 showed a prevalence for granulomatous polyangiitis (GPA) of 210/1 million and for microscopic polyangiitis (MPA) of 46/1 million, corresponding to a prevalence of 256/1 million for AAV overall. Based on these data, Hellmich et al. estimate that approximately 17,500 people live with AAV (GPA and MPA) in Germany [21]. A recent meta-analysis showed a pooled AAV prevalence of 198 (95%CI:187–210)/1 million for all international studies, with very heterogeneous prevalences in the individual studies (44.8 to 421/1 million). Other studies also showed an increase in AAV prevalence over the years [45]. If we continue to calculate the increase in the Lübeck survey from 2006 onwards, the prevalence estimate of AAV by Hellmich et al. appears to be consistent with the data from northern Germany. We therefore estimate there to be about 18,000 affected persons based on the population of 2021.

### Estimation of the total number of people with an inflammatory rheumatic disease in Germany

**Fig. 2:**
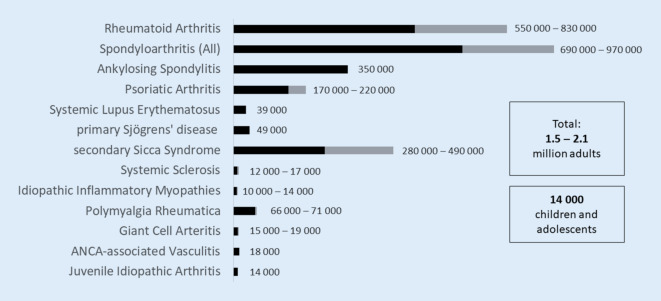
Estimated number of people with an inflammatory rheumatic disease in Germany based on the population of 2021

In 2016, we estimated that approximately 2% of the adult population was affected by an inflammatory rheumatic disease, which corresponded to a number of approximately 1.45 million affected persons [1]. Prevalence estimates from the literature have increased across diseases since then, which seems plausible given the increased life expectancy, decreased mortality, and improved early diagnosis. Since 2014 (data from our last estimate), the proportion of people over 80 has increased by 23% from 5.6% to 7.3% of the population in Germany. Among 60–80-year-olds, there was an increase of 4.4 to a total of 22% of the population [26], which is certainly associated with an increase in the prevalence of chronic inflammatory rheumatic diseases. We therefore estimate today, based on the available literature, that approximately 2.2 to 3.0% of the adult population is affected by an inflammatory rheumatic disease, corresponding to approximately 1.5 to 2.1 million adults (Fig. 2).

### Inflammatory rheumatic diseases in children and adolescents

For children and adolescents, data on juvenile idiopathic arthritis (JIA) are available from the German Health Care Atlas. Here, 73.4/100,000 children and adolescents were diagnosed with JIA in 2009 and 101.5/100,000 in 2015 [22]. In adolescents between 16 and 18 years of age, the prevalence of a JIA diagnosis in claims data of BARMER ranged from 0.11% in 2008 to 0.13% in 2009 and 2010 (cumulative) [23]. Data from the UK in 2018 show a significant difference in prevalence rates when JIA diagnosis codes were used (56.3 [95%CI 53.2–59.6]/100,000) or when clinically validated cases were used (30.6 [95%CI 27.9–33.4]/100,000, age-standardized 43.5/100,000) [46]. The discrepancies in the prevalence estimates of Costello et al. illustrate the uncertainty inherent to the evaluation of claims data for prevalence estimates. Consistent incidence rates of a JIA diagnosis in Germany [22] and also in Denmark [47] over time indicate that the proportion of children and adolescents with JIA is largely stable. For JIA, we stick to the 2016 estimate that approximately 1 in 1000 children are affected by JIA. With 13.9 million children and adolescents < 18 years of age in the German population in 2021 [26], this corresponds to approximately 14,000 children and adolescents. There are no data from Germany for juvenile SLE, juvenile myositides, and vasculitides. For the subgroup of juvenile PsA, the prevalence in the study of Reinhardt et al. was 0.01% [13].

## Practical conclusion

The systematic literature review on the prevalence of inflammatory rheumatic diseases in Germany shows an increase in prevalences in many studies compared to previous evaluations. Almost all studies are based on claims data and all studies have a moderate to high risk of bias. In claims data, only diagnoses and not the existing disease status are documented; furthermore, erroneous and multiple or overlapping coding makes it difficult to reliably determine prevalence. In the absence of multistage population studies, the available data are the only available but also an uncertain source for prevalence estimates. Based on these data, we estimate that today about 2.2 to 3% of adults in Germany have an inflammatory rheumatic disease and 0.1% of children and adolescents have juvenile arthritis, which corresponds to 1.5–2.1 million adults and about 14,000 children and adolescents, respectively.

### Supplementary Information

Strategy for the systematic literature search on the prevalence of inflammatory rheumatic diseases in Germany

## Declarations

For this article no studies with human participants or animals were performed by any of the authors. The studies described in this report are subject to the ethical guidelines specified in the respective reports.

The supplement containing this article is not sponsored by industry.
