# Supplementary material for: Systematic review to estimate the prevalence of inflammatory rheumatic diseases in Germany
Source: Z Rheumatol. 2023 Feb 7;83(Suppl 1):20–30. doi: 10.1007/s00393-022-01302-5 (PMC10879227; doi:10.1007/s00393-022-01302-5)

Search strategy for systematic literature search on the prevalence of inflammatory rheumatic diseases in Germany

PubMed/Medline:

("prevalence"[MeSH Terms] OR "prevalence"[Title/Abstract] OR "frequencies"[Title/Abstract]) AND ("rheumatic disease*"[Title/abstract] OR "rheumatoid arthritis"[Title/Abstract] OR “ankylosing spondylitis” [Title/abstract] OR “spondyloarthritis” [Title/abstract] OR “juvenile arthritis” [title/abstract] OR "systemic lupus erythematosus” [Title/abstract] OR "polymyalgia rheumatica"[Title/abstract] OR "psoriatic arthritis"[Title/abstract] OR "myositis"[Title/abstract]) OR "giant cell arteritis"[Title/abstract] OR "sjogren*"[Title/abstract] OR "ANCA-associated vasculitis"[Title/abstract]) AND ("german"[Title/Abstract] OR "Germany"[Title/Abstract]) AND 2014/01/01:2022/10/31[Date - Publication]

Flow chart

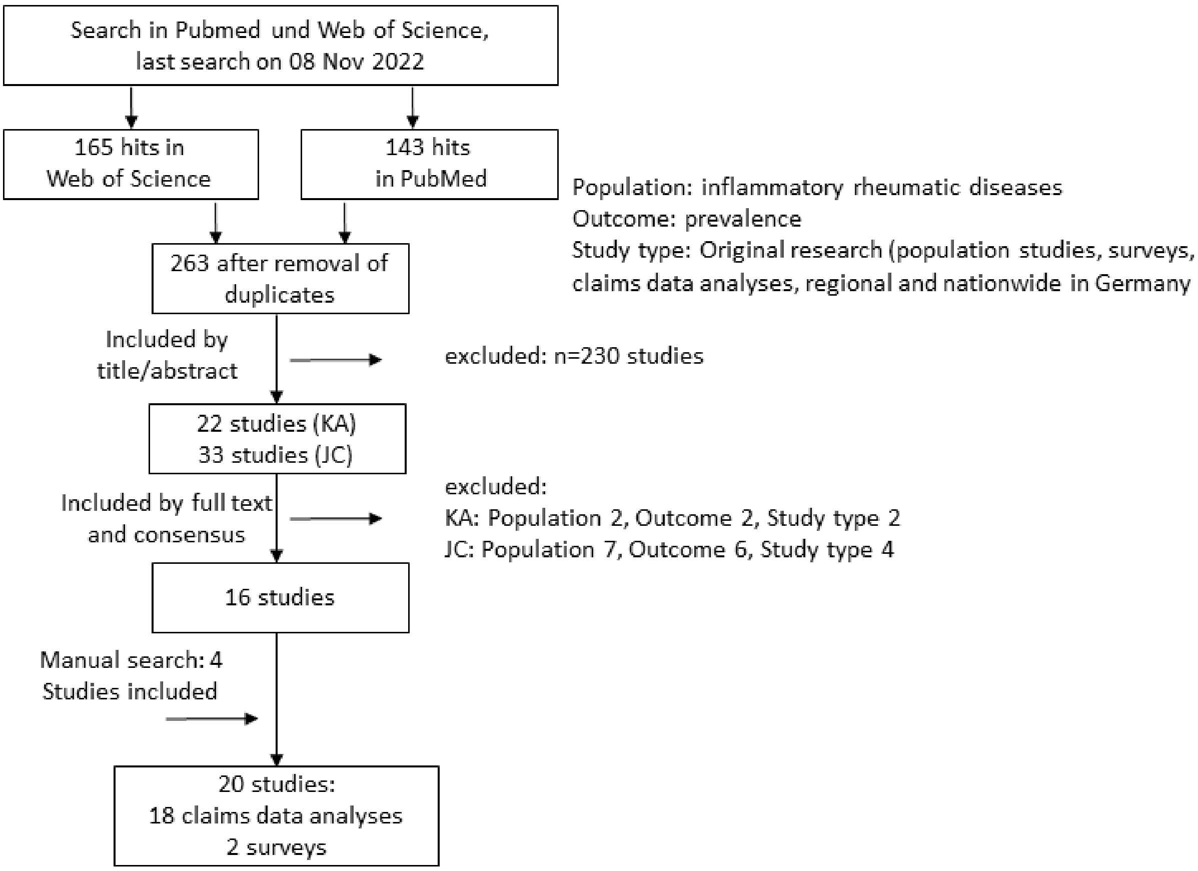

Supplement: Supplementary file 1 — Strategy for the systematic literature search on the prevalence of inflammatory rheumatic diseases in Germany [file 393_2022_1302_MOESM1_ESM.pdf]
